# Confidence in auditory perceptual completion

**DOI:** 10.1093/nc/niaf018

**Published:** 2025-08-15

**Authors:** Cemre Baykan, Pascal Mamassian, Alexander C Schütz

**Affiliations:** Philipps-Universität Marburg, Fachbereich Psychologie, AG Sensomotorisches Lernen, Gutenbergstr. 18, Marburg, 35032, Germany; Laboratoire des Systèmes Perceptifs, Département d’Études Cognitives, École Normale Supérieure, PSL University, CNRS, 24 rue Lhomond, Jaurès building, 2nd floor, Paris, 75005, France; Philipps-Universität Marburg, Fachbereich Psychologie, AG Sensomotorisches Lernen, Gutenbergstr. 18, Marburg, 35032, Germany

**Keywords:** auditory continuity illusion, temporal induction, metacognition, auditory confidence

## Abstract

Previous studies examining confidence in perceptual completion in vision showed that observers can be unaware of missing sensory information and be even more confident in perceptually completed stimuli than veridical stimuli. In the current study, we aimed to investigate if auditory filling-in mechanisms would result in similar confidence biases. In two separate experiments, participants listened to continuous (uninterrupted) or discontinuous (interrupted) tones that were accompanied by noise. We examined confidence for continuity-discontinuity decisions by collecting confidence ratings (Experiment 1) and forced-choice confidence judgments (Experiment 2). Participants reported the interrupted sounds with masking noise more as uninterrupted, showing auditory filling-in. Confidence ratings in the first experiment followed response consistency. Forced-choice confidence judgments in the second experiment showed that participants were not able to distinguish the filled-in stimulus from a continuous stimulus with similar masking noise. Most importantly, there was no clear preference for a veridical compared to a perceptually completed stimulus. These results, extending findings from the visual modality, are the first to demonstrate that listeners are unaware of auditory filling-in and trust filled-in information almost as much as veridical information in the auditory sense.

## Introduction

Sensory information about our environment is generally not seamless and complete, but full of gaps and interruptions. For instance, objects or scenes can be partially occluded or hidden from plain sight, and sounds can be masked temporarily by background noise. In such situations, our brain “fills in” the missing sensory information, constructing a complete perception (Kanizsa 1985; for reviews, see Weil and Rees 2011; Nanay 2018; Thielen et al. 2019). In visual perception, this ability has been best illustrated by amodal completion: when parts of an object are occluded by another object, observers perceive the occluded object in its completeness (Michotte et al. 1964; Kanizsa 1979). Furthermore, auditory studies have demonstrated that perceptual completion or filling-in is not limited to vision, as humans perceive sounds in continuation even when they are interrupted or masked by noise (Warren 1970; Warren et al. 1972). Perceptual completion not only allows us to fill in distal gaps in information that are caused by properties of the environment, but also proximal gaps that are caused by properties of our own sensory systems. Perceptual completion occurs in the blind spot (Ramachandran 1992) and in the foveal rod scotoma (Gloriani and Schütz 2019). Remarkably, in some cases of visual completion, observers are even unaware of filling-in and overconfident about the inferred or filled-in information in vision (Ehinger et al. 2017; Gloriani and Schütz 2019; Men et al. 2023). It is, however, not yet known whether humans are also confident about filled-in (inferred) information in the auditory sense.

Perceptual experiences are accompanied by metacognitive processes that are acquired through judgments of one’s own cognitive and affective states (Fernandez-Duque et al. 2000; Fleming and Lau 2014). Confidence judgment, as a part of metacognitive processes, refers to an estimation of certainty of one’s own perceptual decisions (Mamassian 2016). Usually, humans are good at providing accurate decision-confidence judgments, and their confidence reports typically indicate a positive relationship with correct perceptual decisions (Weil et al. 2013; Desender et al. 2019). However, there are also circumstances where confidence is dissociated from actual perceptual performance. For instance, earlier studies have found that observers trust inferred information more than veridical sensory information in vision (Ehinger et al. 2017; Gloriani and Schütz 2019). Ehinger et al. (2017) showed that individuals choose stimuli presented inside the blind spot more often as continuous compared to stimuli presented outside the blind spot, implying higher reliance on the inferred relative to the sensory information. In another study, Gloriani and Schütz (2019) asked participants to select a continuous stimulus between a central (foveal) and a peripheral stimulus under both photopic (light-adapted) and scotopic (dark-adapted) viewing conditions. Although information is not veridical, but filled-in in central vision under scotopic viewing, they found that individuals prefer a central stimulus more often than a peripheral stimulus in a confidence task under both photopic and scotopic viewing conditions, showing that observers trust filled-in information. Altogether, these studies suggest that human observers fail in their metarepresentation of the filled-in stimulus, exhibiting metacognitive distortions in the visual modality.

Similar to the visual system’s filling-in, the auditory system restores or completes missing sensory information (Warren 1970; Warren et al. 1972; Bregman 1994). For example, this phenomenon has been shown in the “auditory continuity illusion”: when pure tones are interrupted with some parts having no auditory input but being masked by noise, human listeners perceive the tones as uninterrupted or continuous through the noise (Vinnik et al. 2010). This illusion has been found to be very robust and observed for a variety of sounds involving tones, melodies, and speech (Bashford Jr et al. 1988; Bregman 1994; Miller et al. 2001; Micheyl et al. 2003; Riecke et al. 2007; McWalter and McDermott 2019). Micheyl et al. (2003) reported that participants perceive a target sound as continuous when the silent gap between two tones is filled with noise that covers the same frequency band (on-frequency). In another study, Shahin et al. (2009) showed that listeners perceive words as if they are uninterrupted even though a segment of the target words is replaced by white noise. Perceptual completion in the auditory sense has been suggested to show similarities to visual amodal completion (Petkov and Sutter 2011; Nanay 2018). A good example of visual amodal completion is seeing an animal behind a picket fence as a complete object (Nanay 2018). The ability to complete the missing information depends on the good continuation of the partially occluded object behind the occluder and the visible properties of the occluder, such as its size or the clarity of the contour (Palmer et al. 2007; Scherzer and Faul 2019). This is analogous to auditory completion because auditory or temporal induction of a signal only occurs when several conditions are met. First, the signal needs to have a sufficient duration around the noise; the noise needs to fill the gap in the signal both temporally and spectrally (without any gap); and finally, the noise needs to have an adequate sound pressure (Bregman 1994; Petkov and Sutter 2011). Despite profound similarities between auditory and visual completion, there is a fundamental difference: while the auditory system fills gaps in the temporal domain, the visual system produces primarily filling-in in the spatial domain.

Here, we aimed to investigate perceptual discrimination performance for auditory stimuli with and without completion, and the confidence for these decisions. To this end, in two separate experiments, we presented continuous and discontinuous tones, masked by a noise burst, to participants and asked them to make judgments on stimulus continuity and response confidence. In Experiment 1, participants were presented with a single stimulus and were asked to make a continuity report followed by a confidence judgment on a 2-point rating scale (high versus low confidence). In this experiment, we collected confidence ratings and perceptual decisions on every trial to analyze the relationship between perceptual performance and confidence judgments (Mamassian 2016). However, confidence rating measures have two main drawbacks: being sensitive to individual differences (e.g. how participants generate or use the confidence ratings internally) and potentially confusing participants to report the perceived strength of sensory information (its “visibility”) rather than confidence itself (Aitchison et al. 2015; Mamassian 2016; Rausch and Zehetleitner 2016). Therefore, we employed a confidence forced-choice method in Experiment 2, where participants heard two sequential sounds and made their continuity judgments, and then, they selected for which stimulus judgment they felt more confident (Mamassian 2020).

In both experiments, we examined the relationship between the discrimination performance and the metacognitive confidence. In line with previous studies (Micheyl et al. 2003; Vinnik et al. 2010), we expected that individuals would perceive discontinuous tones with on-frequency noise more as continuous, experiencing perceptual filling-in. We predicted that if they trust filled-in information in auditory completion, they should report high confidence, but if they are aware of filling-in in a particular trial and do not trust the inferred information, they should respond with low confidence to this stimulus condition. Consistent with the former, our results showed that participants objectively perceived discontinuous tones with on-frequency noise more as continuous, and crucially, they reported this judgment with a high level of confidence.

## Materials and Methods

### Participants

Twenty-two and 20 participants with no self-reported hearing impairment took part in Experiments 1 and 2, respectively. Each participant received a monetary reward of 8 euros for their participation. All participants provided written informed consent before the experiments started. We excluded two participants from further analyses in Experiment 1 given that their perceptual accuracy for all conditions were around 50% chance level, suggesting unreliable performances for these two participants. As a result, we entered the data of 20 participants (10 females; age range: 19–45 years; mean age of 26.9 years) to the main analysis in Experiment 1 and 20 participants (12 females; age range 17–45 years; mean age of 26.6 years) in Experiment 2. Sixteen of the Experiment 1 participants also participated in Experiment 2. All participants were naive to the purpose of the study. The experiments were approved by the ethics committee of the Psychology Department at the University of Marburg (proposal number 2021-71k).

### Apparatus and stimuli

All auditory stimuli were generated using the Psychophysics Toolbox on MATLAB (Version R2023a; The Mathworks, Inc.) at a sampling rate of 44.1 kHz and were played with a 16-bit resolution soundcard. The stimuli were presented binaurally via headphones (Sennheiser HD 600, Sennheiser, Wedemark, Germany). The tones and the noises were generated separately, with the sound pressure level (SPL) of 60 and 69 dB (A), respectively, measured at the eardrum using the miniDSP Earphone Audio Response System device (miniDSP, Hong Kong, China). For the continuous tone, the tone and the noise were overlapping, while for the discontinuous tone, the noise was placed in a silence gap. Due to the logarithmic scale of decibel levels, the total SPL of the combination of the continuous tone and the overlapping noise was still close to 69 dB. The experiments were conducted in a dimly lit and sound-attenuated experimental room.

**Figure 1:**
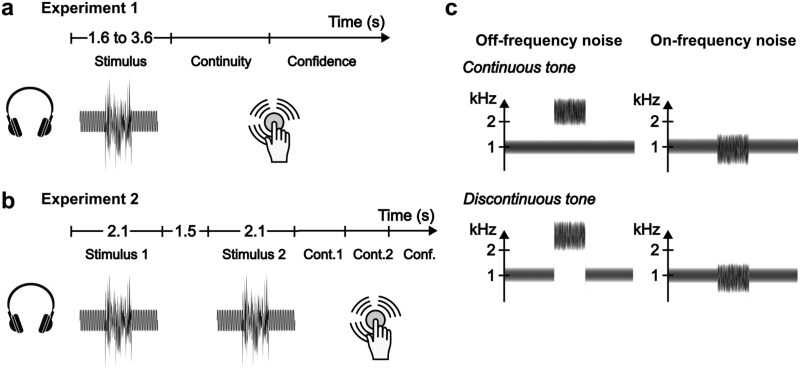
(a) Experiment 1: the stimulus was presented for 1.6−3.6 s. Then, the participants made a continuity decision and lastly, a response confidence judgment. (b) Experiment 2: two stimuli were presented for 2.1 s with an interstimulus interval of 1.5 s. Then, the participants made two continuity decisions regarding the first and second stimuli, and one confidence judgment to report which continuity decision they had more confidence in. (c) Spectrograms of the target stimuli used in both experiments: a continuous and a discontinuous tone with an off-frequency and on-frequency noise.

Similar to previous studies that examined the continuity illusion in auditory signals (Vinnik et al. 2011, 2012), we used either continuous or discontinuous sounds of pure sine waves (1007 Hz) as target stimuli (Fig. 1c). All target stimuli, except for the control trials in Experiment 2, had accompanying noise bursts, which could either be at 900 Hz (masking/on-frequency noise) or 2425 Hz (spectrally dissimilar/off-frequency noise) with a bandwidth of 600 Hz. Noise bursts were superimposed on the target stimulus centrally: overlapping with the tone in the continuous stimulus and with the silence gap in the discontinuous stimulus. In both conditions, the tone was noise-free for the first 800 ms and the last 800 ms. Both tones and noises had 7-ms cosine-squared onset and offset rampings. Crucially, in the discontinuous tone conditions, the ramps of the tones and the noises were overlapping to prevent an abrupt discontinuity signal.

### Procedure

The experiments consisted of two variants of tone (continuous versus discontinuous) and two variants of noise (on-frequency versus off-frequency noise). The noise (and gap) durations were 50, 100, 200, 500, or 2000 ms in Experiment 1, and 500 ms in Experiment 2.

Before the main experiment, participants performed a practice sequence consisting of eight trials, where each condition with a 2000 ms noise duration was presented two times. Practice trials started with a text presentation of either “continuous tone” or “discontinuous tone” to inform the participants which target stimulus they would be listening to. After the sound presentation, they did not give any response. During the main testing, the monitor was switched off to prevent any noise in the testing room. Participant responses were collected via a five-button response box. Participants initiated a trial pressing the “central” button, and gave continuity decision responses using upper right–left buttons and confidence ratings using lower right–left buttons. In both experiments, the mapping of the continuous-discontinuous and high-confident or low-confident responses was counterbalanced across participants: half of the participants responded with the left buttons to continuous and high confident, while the other half responded with the left buttons to continuous and low confident.

In Experiment 1 (Fig. 1a), participants listened to a single stimulus and made one decision on stimulus continuity and one judgment on their response confidence. In Experiment 2 (Fig. 1b), participants heard two sequential sounds separated by a 1.5-s gap, and they were asked to make continuity decisions of these two sounds and a confidence judgment for which continuity decision they were more confident. Upon pressing a response button, they heard a vocalization of the response they made.

There were six paired conditions for the sequential sounds in Experiment 2, with two making a comparison of tone conditions: D-off versus C-off (discontinuous tone with off-frequency noise versus continuous tone with off-frequency noise) and D-on versus C-on (discontinuous tone with on-frequency noise versus continuous tone with on-frequency noise); two making a comparison of noise conditions: D-on versus D-off (discontinuous tone with on-frequency noise versus discontinuous tone with off-frequency noise) and C-on versus C-off (continuous tone with on-frequency noise versus continuous tone with off-frequency noise); and two making a comparison of mixed conditions: D-off versus C-on (discontinuous tone with off-frequency noise versus continuous tone with on-frequency noise) and D-on versus C-off (discontinuous tone with on-frequency noise versus continuous tone with off-frequency noise). The presentation order of the sequential sounds was counterbalanced for each paired condition.

Experiment 1 consisted of 400 trials, each condition (tone continuity, noise frequency, and noise duration) was repeated 20 times. Experiment 2 had 256 trials of which 16 were control trials (where no noise was presented) and 240 were main trials: each condition (tone continuity, noise frequency, and pair type) was repeated 20 times and the presentation order of the stimuli across the experiment was counterbalanced. In control trials, one of the two stimuli did not have an accompanying noise burst (a pure continuous or discontinuous sine-wave tone), while the other stimulus was chosen from one of the stimulus conditions (Fig. 1c), and their presentation order was counterbalanced.

### Statistical analysis

All statistical tests were carried out in R (version 4.3.2; R Core Team 2023). In Experiment 1, proportion correct and confidence ratings were analyzed using a linear mixed model via *lmerTest* (version 3.1–3) package (Kuznetsova et al. 2017) and using a multiple regression via *stats* (version 3.6.2, R Core Team 2023) package. In Experiment 2, we reported the proportion of correct responses for stimulus discrimination judgments. We calculated the means of each stimulus condition per participant. Similar to a previous study (Gloriani and Schütz 2019), confidence intervals (95%) were bootstrapped using 20 000 bootstrap samples and a bias corrected and accelerated percentile method.

## Results

### Experiment 1

**Figure 2:**
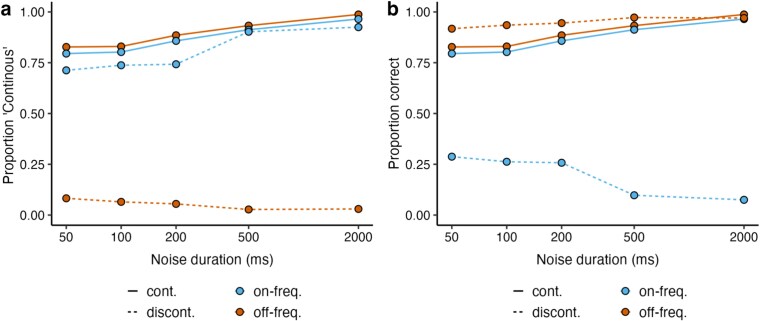
(a) Proportion of “continuous” answers and (b) correct judgments as a function of noise duration. Solid lines indicate continuous stimuli; dashed lines the discontinuous stimuli. Cyan represents on-frequency noise; orange off-frequency noise conditions. Note that the *x*-axis has a logarithmic scale.

In the first task, participants had to categorize tones as continuous or discontinuous. Figure 2a and b shows the proportion of “continuous” responses and correct judgments as a function of noise duration, respectively. In the off-frequency conditions, continuous and discontinuous stimuli were accurately categorized. However, in the on-frequency conditions, both continuous and discontinuous stimuli were reported as continuous, indicating that participants experienced filling-in in this condition, in line with previous work (Vinnik et al. 2011, 2012). To analyze the results statistically, we employed a linear mixed model with fixed factors of noise duration, tone type (continuous as 0, discontinuous as 1), and noise type (off-frequency as 0, on-frequency as 1), as well as the interaction term of tone and noise type. The results revealed that response accuracy increased with noise duration ($\beta$ = 0.02, SE = 0.005, *t* = 3.65, *P* < .001). There was a significant effect of tone type for off-frequency noise, showing that response accuracy was higher for discontinuous compared to continuous tones ($\beta$ = 0.06, SE = 0.01, *t* = 5.45, *P* < .001). The significant interaction between tone and noise types indicates that response accuracy also differed between tone types for on-frequency noise but in an opposite direction ($\beta$ = −0.73, SE = 0.01, *t* = −50.43, *P* < .001). This result indicates that the proportion correct for on-frequency noise conditions decreased for discontinuous relative to continuous tones approximately by 0.67 (−0.73 + 0.06). In addition, the response accuracy was lower in the on-frequency compared to off-frequency noise for continuous tones ($\beta$ = −0.03, SE = 0.01, *t* = −2.55, *P* = .01). Taking the significant interaction term into account, the results also point out a significant difference between on-frequency and off-frequency noise for discontinuous tones of around 0.76 (−0.73–0.03).

**Figure 3:**
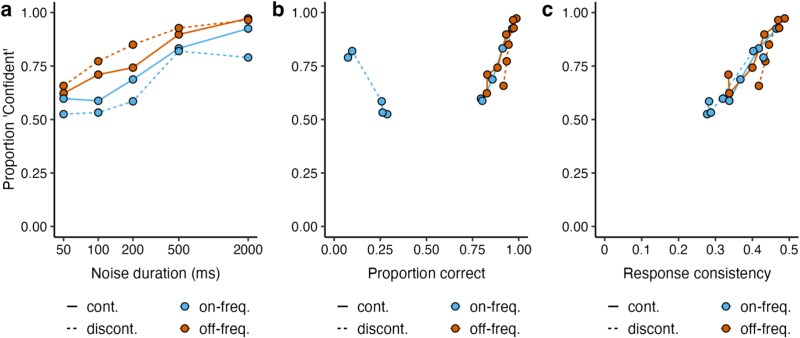
(a) Confidence ratings as a function of noise duration. Note that the *x*-axis has a logarithmic scale. Confidence ratings as a function of (b) proportion correct and (c) consistency of the continuity responses. Solid lines indicate continuous stimuli; dashed lines discontinuous stimuli. Cyan represents on-frequency noise; orange off-frequency noise conditions.

In the second task, participants had to rate if their confidence about their continuity decision in the first task was high or low. Figure 3a shows the proportion of “confident” ratings as a function of noise duration and indicates that high confidence grows with noise duration. If participants were aware of the filling-in with the on-frequency noise in a particular trial and their inability to discriminate accurately between a continuous and a discontinuous tone with on-frequency noise, one would expect that they always responded with a low-confidence rating whenever there was an on-frequency noise. However, this was not the case, because the proportion of high-confident responses was above 50% for both off-frequency and on-frequency noise. We employed a separate linear mixed model with fixed factors of noise duration, tone type (continuous as 0, discontinuous as 1), and noise type (off-frequency as 0, on-frequency as 1), as well as the interaction term of tone and noise type. The results revealed that confidence ratings increased with noise duration ($\beta$ = 0.14, SE = 0.006, *t* = 22.74, *P* < .001). There was a significant effect of tone type for off-frequency noise, showing that confidence ratings were higher for discontinuous compared to continuous tones ($\beta$ = 0.05, SE = 0.01, *t* = 3.70, *P* < .001). The significant interaction between tone and noise types indicates that confidence ratings also differed between tone types for on-frequency noise condition but in an opposite direction ($\beta$ = −0.12, SE = 0.02, *t* = −6.96, *P* < .001). This result shows that the proportion confidence for the on-frequency noise conditions decreased for discontinuous relative to continuous tones approximately by 0.07 (−0.12 + 0.05). In addition, the confidence was lower in the on-frequency compared to off-frequency tone for continuous tones ($\beta$ = −0.06, SE = 0.01, *t* = −5.12, *P* < .001). Taking the significant interaction term into account, the results also point out a significant difference between on-frequency and off-frequency noise for discontinuous tones in proportion confidence at around 0.18 (−0.12–0.06).

Instead of using an estimate of the accuracy of their perceptual decisions to report confidence, participants may have computed the consistency of their decisions. Perceptual consistency refers to the stability of making the same decision for a given stimulus, irrespective of being correct or incorrect (Caziot and Mamassian 2021). Therefore, we decided to analyze confidence either as a function of proportion correct (Fig. 3b) or response consistency (Fig. 3c). We calculated response consistency by calculating the absolute distance of continuity responses to the subjective equality between continuous and discontinuous (i.e. 0.5, given that discontinuous responses were coded as 0, and continuous as 1). We conducted two separate multiple linear regression tests to examine if confidence can be predicted by the variables of either proportion correct or response consistency, noise duration, tone type, and noise type, while we added an interaction term for tone and noise type. In these regression tests, response consistency, proportion correct, and noise duration were normalized within the range of 0 and 1 using min−max scaling, so that the range of these predictors was the same. The first regression test, which included the predictors response consistency, noise duration, tone type, and noise type, had a better goodness of fit, adjusted *R*^2^ = 0.50, *F*(5, 394) = 81.13, *P* < .001, compared to the second regression test, which included the same predictors, but proportion correct instead of response consistency, adjusted *R*^2^ = 0.24, *F*(5, 394) = 26.03, *P* < .001. The results of the first regression revealed that confidence ratings increased with response consistency ($\beta$ = 0.88, SE = 0.06, *t* = 14.98, *P* < .001) and noise duration ($\beta$ = 0.08, SE = 0.01, *t* = 6.60, *P* < .001). The categorical variables were dummy coded: continuous tone as 0, discontinuous tone as 1; off-frequency noise as 0, on-frequency noise as 1. The continuous tone with off-frequency noise significantly predicted the confidence ratings ($\beta$ = 0.40, SE = 0.03, *t* = 13.83, *P* < .001) and that intercept was significantly higher in the discontinuous tone with off-frequency noise ($\beta$ = 0.79, SE = 0.06, *t* = 14.15, *P* < .001). The model showed no additional significant evidence for continuous tone with on-frequency noise ($\beta$ = −0.04, SE = 0.03, *t* = −1.58, *P* = .17), suggesting a comparable effect of continuous tone with on- and off-frequency noise conditions on confidence ratings. There was a significant interaction between tone and noise type ($\beta$ = −0.81, SE = 0.06, *t* = −13.87, *P* < .001), indicating a significant effect of discontinuous tone with on-frequency noise. Overall, the findings of this regression suggest that confidence ratings were strongly predicted by response consistency.

### Experiment 2

To compare on- and off-frequency conditions more directly, we used a confidence-forced-choice paradigm in Experiment 2, where listeners had to directly compare their confidence across two consecutive perceptual decisions issued from two different stimuli.

We assessed the reliability of participants’ performance by measuring their perceptual responses in control trials that contained a stimulus without any noise. The result showed that participants responded to the pure continuous or discontinuous sine wave tones in control trials with perfect accuracy (100% for 15 participants, 98% across all 20 participants). Moreover, participants preferred the judgments of pure tone over the judgments of tones with noise as more confident at 82.5% of all control conditions.

**Figure 4:**
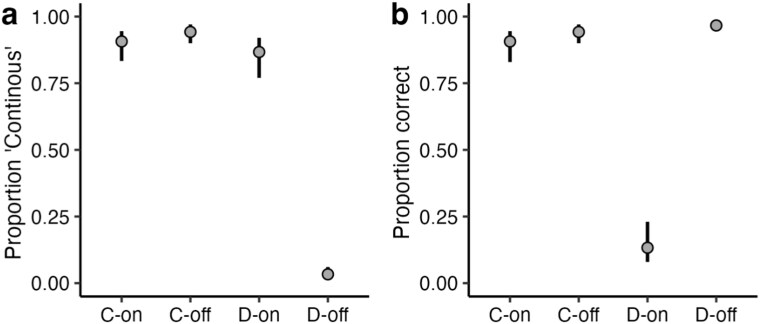
(a) Proportion of “continuous” answers and (b) correct judgments. Error bars are bootstrapped 95% CIs.

Figure 4a and b shows the proportion of “continuous” responses and correct judgments for stimuli with on- and off-frequency noise. We calculated the proportion of correct decisions per participant for different stimulus conditions. Participants performed better than 50% chance level for the continuous stimulus when masked with on-frequency noise (90.7%, CI_95%_[83, 94.5]) and with off-frequency masking noise (94.3%, CI_95%_[89.9, 96.5]). This perceptual performance was increased at the discontinuous stimulus with off-frequency masking noise (97.7%, CI_95%_[94.5, 97.8]). Participants had the worst performance for the discontinuous stimulus when masked with on-frequency noise (13.3%, CI_95%_[8.1, 22.9]), i.e. they were strongly biased to respond “continuous” in this condition, thus replicating our results from Experiment 1.

**Figure 5:**
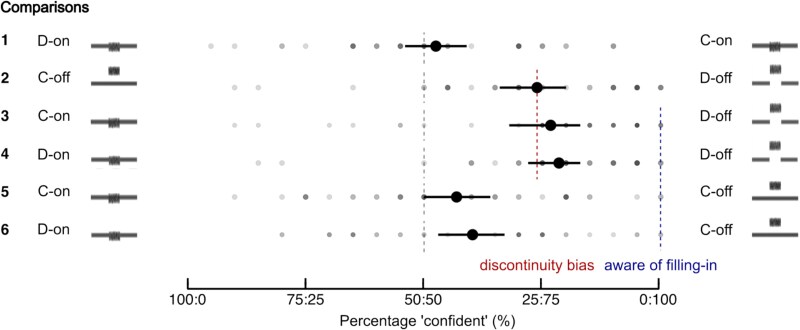
Confidence judgments in the forced-choice paradigm. Shaded dots represent individual data; solid dots the average across participants. Error bars denote bootstrapped 95% CIs. The gray dashed line at 50% represents a 50% unbiased selection if participants were not aware of filling-in in a trial and had no bias for inferred information. If participants were aware of the filling-in for the on-frequency noise, a zero preference for the target stimuli with on-frequency noise was expected when they were compared against stimuli with off-frequency noise (the last four comparisons; blue dashed line at 100%). A global bias for the discontinuous stimulus was observed (red dashed line near 75%), as shown by the mean preference for discontinuous over continuous stimuli with off-frequency noise (second row).

Figure 5 shows the percentage of “confident” responses given in the forced-choice paradigm. When the participants made a forced-choice confidence judgment between continuous and discontinuous stimuli with both having an on-frequency noise, their selection was almost at 50% unbiased level (row 1, continuous-on | discontinuous-on: 52.5%, CI_95%_[45.8, 59]). This is further evidence that participants were not able to discriminate between a discontinuous and a continuous stimulus with on-frequency noise. In cases where they were able to discriminate between continuous and discontinuous stimuli, they showed a preference for discontinuous over continuous stimuli, as shown by the comparison between a continuous and a discontinuous stimulus with off-frequency noise (row 2, discontinuous-off | continuous-off: 73.9%, CI_95%_[66.1, 80]) and the comparison between a continuous stimulus with on-frequency noise and a discontinuous stimulus with off-frequency noise (row 3, discontinuous-off | continuous-on: 76.8%, CI_95%_[68, 82.8]), and also for the comparison between a discontinuous stimulus with on-frequency noise (mostly perceived as continuous) and a discontinuous stimulus with off-frequency noise (row 4, discontinuous-off | discontinuous-on: 78.5%, CI_95%_[71.5, 83.3]). Finally, when comparing two stimuli that were perceived as continuous between noise conditions, there was a slight preference for the off-frequency noise over the on-frequency noise. There was a trend for the comparison between two continuous stimuli with on- and off-frequency noise (row 5, continuous-off | continuous-on: 56.9%, CI_95%_[49.4, 63.8]) and a significant effect for the comparison between a discontinuous stimulus with on-frequency noise and a continuous stimulus with off-frequency noise (row 6, continuous-off | discontinuous-on: 60.3%, CI_95%_[53.4, 67]). These results show that participants were not aware of the masking by the on-frequency noise and that they trusted the filled-in percept in that condition.

## Discussion

We tested in two experiments whether humans are overconfident about filled-in information in the auditory modality. To this end, we presented continuous or discontinuous tones that were accompanied by either an on-frequency (masking) or an off-frequency (spectrally dissimilar) noise. In Experiment 1, participants listened to a single stimulus and made a confidence rating after the perceptual judgment, whereas in Experiment 2, they listened to two consecutive stimuli and made a forced-choice confidence judgment after the two perceptual decisions. In both experiments, we presented to the participants an example of each stimulus condition to make them aware of the auditory filling-in phenomenon before running the experiments. Our results show that individuals experienced filling-in of discontinuous (interrupted) tones when they were masked by a masking noise in both experiments, reflecting their inability to recognize the filling-in in a given trial. We expected that participants should be less confident in those masking conditions if they were aware of the filling-in. Contrary to that prediction, confidence ratings primarily depended on response consistency for both, on- and off-frequency noise in Experiment 1. This is in line with the findings of Caziot and Mamassian (2021), suggesting that confidence reflects an estimate of the reliability of one’s perceptual decision. Furthermore, confidence judgments in Experiment 2 showed that individuals were unaware of the filled-in information and had no clear preference for the off- compared to the on-frequency noise. Instead, they had a confidence bias for discontinuous over continuous stimuli.

Our first hypothesis was that perceptual decisions of individuals would reflect auditory filling-in. Auditory induction or restoration posits that listeners capably complete the missing segments of sounds (Warren et al. 1972). Interestingly, this is not just a perceptual omission of the missing information: silence gaps are not omitted but “heard” similarly to sounds (Goh et al. 2023). For example, Goh et al. (2023) showed that auditory illusions, e.g. one-is-more illusion (Yousif and Scholl 2019), occur to silence gaps in the same way as to sounds. Furthermore, earlier studies suggested that auditory induction is an actual completion on a neural level, as shown by activity in the primary auditory cortex for simple tones (Petkov et al. 2007; Riecke et al. 2007) and higher areas for more complex sounds (Riecke et al. 2012). Auditory induction occurs when the tone and noise overlap in frequency and the signal-to-noise ratio is higher (Warren et al. 1988; Bregman et al. 1990; Kluender and Jenison 1992; Riecke et al. 2008), even in the absence of attention (Micheyl et al. 2003). In line with this, earlier studies reported that listeners judge interrupted tones masked with extraneous sounds more as uninterrupted (Micheyl et al. 2003; Vinnik et al. 2010). Here, we found consistent results with these studies given that in both experiments, participants reported the discontinuous tones with a masking noise more as a continuous tone. Moreover, we found that this continuity illusion increases with increasing noise duration, in line with previous findings (Riecke et al. 2008; Vinnik et al. 2011).

For perceptual confidence, we hypothesized that if listeners are aware of the missing sensory information and their perceptual completion, they should not trust the completion and respond with low confidence. However, we found that listeners responded to the filled-in stimulus, i.e*.* a discontinuous tone with on-frequency noise, with high confidence. This was shown by high confidence ratings above unbiased selection level for the filled-in stimulus in Experiment 1, as well as a comparable preference of on-frequency and off-frequency noise conditions in Experiment 2. These findings complement visual studies showing that humans have high confidence for inferred stimuli in vision as elicited by filling-in of retinal scotomata such as the blind spot or the foveal rod scotoma (Ehinger et al. 2017; Gloriani and Schütz 2019). Importantly, however, individuals had a higher preference for inferred or filled-in stimuli over veridical visual stimuli in those studies (Ehinger et al. 2017; Gloriani and Schütz 2019), whereas, in our auditory data listeners did not prefer filled-in stimuli (on-frequency noise) over veridical stimuli (off-frequency noise). This difference between auditory completion and the completion of visual scotomata might be related to the origin of the missing information: in our auditory paradigm, filling-in occurs due to external (environmental) reasons, and in the mentioned visual studies (Ehinger et al. 2017; Gloriani and Schütz 2019), perceptual completion happens as a result of internal (anatomical) limitations. Given this difference, filled-in representations might vary in their noise level and/or these perceptual completions might arise at different processing stages. Indeed, previous studies indicated that performance over the blind spot can be better than the intact retina, e.g. in a Vernier task (Crossland and Bex 2009), and reaction times are faster for selecting a stimulus presented over the blind spot than at an equidistant intact nasal location (Ehinger et al. 2017). Consequently, it is possible that perceptual filling-in, which occurs due to internal reasons in vision, gives rise to filled-in representations that are less prone to noise or result from more automatic mechanisms, hence observers trust that inferred information more compared to its auditory counterpart. It is still an open question to what extent auditory induction and filling-in at retinal scotomata reflect similar or distinct processes; future studies might directly compare these examples of perceptual completion.

Our results further indicated a general bias for selecting the judgments of discontinuous tones as more trustworthy (a higher confidence). This observation corroborates studies showing a larger confidence for stimulus presence (in our case, the presence of a gap) rather than absence (Kanai et al. 2010; Meuwese et al. 2014; Mazor et al. 2020, 2021; Kellij et al. 2021). Meuwese et al. (2014) found that observers have higher metacognitive abilities in a categorization task than in a detection task, and this resulted from “correct rejection trials” where participants correctly reported an absence of a target, suggesting that positive evidence gained from a distractor (in categorization) increases confidence relative to the case of the absence of such evidence (in detection). This also aligns with the earlier findings that showed faster reaction times for stimulus-present trials (Mazor et al. 2020, 2021). Our data demonstrate higher confidence for discontinuous stimulus judgments compared to judgments of continuous tones or discontinuous tones with a masking noise (which are also perceived as continuous tones). It is likely that a spectrally clear temporal gap in a tone was analogous to a target presence; hence, when participants detected a discontinuity (target), this increased their confidence.

In conclusion, our results show that interrupted tones when masked by extraneous sounds were perceived to be uninterrupted. Combining the findings from two experiments, we observed that listeners were unaware of filling-in in the auditory domain, and they trusted these filled-in stimuli in their confidence judgments. Moreover, the data showed that listeners had a general bias for selecting the judgments of discontinuous stimuli as more trustworthy. Our results indicate that humans are deceived by filled-in information in the auditory sense, as evidenced by their equal trust for stimuli that are filled-in and stimuli that are perceived veridically. This is in line with the confidence findings from the visual modality, although in these cases, observers reported even higher confidence for filled-in compared to veridical information (Ehinger et al. 2017; Gloriani and Schütz 2019; Men et al. 2023). It remains to be seen the degree to which these findings extend to other cases of auditory or visual completion.

## Supplementary Material

stimCoff_niaf018

stimCon_niaf018

stimDoff_niaf018

stimDon_niaf018

## Data Availability

The data supporting the findings of this study and the statistical analysis code used in the manuscript are available at https://doi.org/10.5281/zenodo.15729434. Sample stimuli are provided in Supplementary data.
